# Response Surface Methodology: Optimisation of Antifungal Bioemulsifier from Novel *Bacillus thuringiensis*

**DOI:** 10.1155/2014/423289

**Published:** 2014-10-14

**Authors:** Deepak Rajendran, Ponnusami Venkatachalam, Jayapradha Ramakrishnan

**Affiliations:** ^1^School of Chemical and Biotechnology, SASTRA University, Thirumalaisamudram, Thanjavur, Tamil Nadu 613401, India; ^2^Centre for Research on Infectious Diseases (CRID), School of Chemical and Biotechnology, SASTRA University, Thirumalaisamudram, Thanjavur, Tamil Nadu 613401, India

## Abstract

An antifungal bioemulsifier compound was produced from a novel strain of *Bacillus thuringiensis* pak2310. To accentuate the production and as the first step to improve the yield, a central composite design (CCD) was used to study the effect of various factors like minimal salts (1X and 3X), glycerol concentration (2% and 4%), beef extract concentration (1% and 3%), and sunflower oil concentration (2% and 4%) on the production of bioemulsifier molecule and to optimize the conditions to increase the production. The *E*_24_ emulsification index was used as the response variable as the increase in surfactant production was seen to be proportional to increased emulsification. A quadratic equation was employed to express the response variable in terms of the independent variables. Statistical tools like student's *t*-test, *F*-test, and ANOVA were employed to identify the important factors and to test the adequacy of the model. Under optimum conditions (1X concentration of minimal salts (MS), 2.6% glycerol (v/v), 1% beef extract (w/v), and 2% sunflower oil (v/v)) a 65% increase in yield was produced.

## 1. Introduction

The entire dynamics of the world is based on chemical reactions of big and small molecules alike. In fact, investigation into the origin of life revealed that certain small molecules are the building blocks of even complex organisms. In this world of chemicals, one could easily figure out that certain harmful molecules can be substituted with ecofriendly biomolecules. It is very interesting to note a chemical that is completely synthetic and which performs a function that has its biological analogue too. Say, for example, we can use cellulolytic and ligninolytic enzymes instead of alkali in the paper industry for pulping and processing [1–3] and, similarly, there are so many biomolecules to substitute the synthetic and inorganic chemicals that are at times harmful too. The most used group of chemicals that are present in almost all the products around us are the surfactants or the surface active agents. Surfactants are those chemicals that are capable of lowering the interfacial energy at an immiscible interface and hence promote the mixing and homogenization of the two phases to form microemulsions [4]. There are many emulsions and gels that we use and are in touch with. All these are mostly petroleum derived complex molecules that are sulphonated and hence very difficult to be biologically degraded [5]. To combat this problem, we could use a surfactant or emulsifier of biological origin.

Intense research into new antibiotics has led to the finding of a unique class of compounds altogether. As these molecules have both hydrophobic and hydrophilic regions just like a molecule of any other detergent, it is not surprising that they form micelles and act as detergents. The facts are that these bioemulsifiers, apart from having less toxicity, high stability, and other qualities, also have a very low Critical Micellar Concentration (CMC), which means that only less amount of bioemulsifier is needed to form micelles when compared to the most used commercial surfactants of today like sodium dodecyl sulphate [6].This will reduce the amount of surfactants being added to the environment too, as addition of surfactants directly to any environment would affect the inherent microbiota.

Most of the products like cosmetics, jams, jellies, squashes, and other colloidal products that we use in our daily life are emulsions and all these emulsions contain surfactants. Identification of new and efficient producer strains, strain improvements, and economic downstream processes can only help us commercialise bioemulsifiers. But the myriads of advantages associated with bioemulsifiers as reported by various literatures like biodegradability, lower toxicity, mild production conditions, selectivity, and higher specific activity in a wider range of pH, temperature, and salinity [7] should not be overshadowed by setbacks such as lack of cost effective raw materials or cheap technology for scale up.

The applications of bioemulsifiers can be found in a broad spectrum of industries like food, cosmetic, chemical, and pharmaceutical industries [5–11]. Apart from these industries, the application of bioemulsifiers also reaches many environmental engineering applications such as bioremediation, enhanced oil recovery, and soil washing [12]. Some bioemulsifiers also show high antimicrobial activity [13]. This study is based on a bioemulsifier from* Bacillus thuringiensis* that exhibits significant antifungal activity against the emerging human pathogen,* Fusarium oxysporum* [14–17].

Media composition and production conditions are the two main areas where optimization has to be done as the first step towards commercialisation of any bioproduct. There are several studies where statistical methods have been employed to optimize the physical and physiochemical factors affecting production of bioemulsifier [18, 19]. The scope of this study is to improve the production of the bioemulsifier from the chosen isolate by optimizing the chemical factors or the media composition of the production medium, employing statistical methods. Similarly, response surface methodology was used to find the optimum production and hence antifungal activity of a bioemulsifier from* Bacillus subtilis* by 55% in solid state fermentation experiment [20]. The multivariate response surface methodology has been adopted for optimising the production media as it is less time and resource consuming than the conventional univariate experiments where only one component is varied while the others are fixed [21, 22]. The significant interactions or relative importance of all interactions can be identified easily with only less number of experiments [23–25].

## 2. Materials and Methods

### 2.1. Microorganism

The bioemulsifier producer* Bacillus thuringiensis* pak2310, isolated from diesel contaminated soil with NCBI GenBank accession number JF512478, was grown in nutrient broth supplemented with 1% glycerol. The cell suspensions were stored in 20% glycerol at −80°C.

### 2.2. Medium and Growth Conditions

A loopful of pak2310 was inoculated in the media containing 3% (v/v) glycerol, 1% (w/v) beef extract, and 1X minimal salts (g L^−1^), MgSO_4_ 0.2, CaCl_2_ 0.02, KH_2_PO_4_ 1.00, K_2_HPO_4_ 1.00, NH_4_NO_3_ 1.00, and FeCl_3_ 0.05. The culture was incubated at 37°C and 130 rpm for 12 h. Then 2% of the culture was used as seed inoculum.

### 2.3. Media Optimisation

#### 2.3.1. Preliminary Screening: One Factor a Time Method

The effect of carbon sources (glycerol, sorbitol, and mannitol) at 3% (v/v), organic nitrogen sources (beef extract, yeast extract, and peptone), and inorganic nitrogen sources (NH_4_NO_3_, NaNO_3_, and NH_4_Cl) at 1% (w/v) on growth and emulsification was studied using classical approach. The effect of mineral salts (MS) on growth and emulsification was also studied after the selection of carbon and nitrogen sources by comparing growth and emulsification of cultures of* B. thuringiensis*, with and without 1X MS.

#### 2.3.2. Analytical Methods

The effect of each factor on the growth of the study strain was determined by recording the OD at 600 nm. The antifungal activity was performed by agar well diffusion method. The surface activity was evaluated using the *E*_24_ index as described by Cooper and Goldenberg [10] and oil spread assay as described by de Cássia et al. [26]. Total protein concentration was determined according to Lowry et al., by using bovine serum albumin as the standard.

#### 2.3.3. Central Composite Design

The importance of optimising the media components as one of the crucial steps to increase the yield of bioemulsifier was first shown by Gu et al. [25]. From the results of the previous experiments and from other literature five independent factors were chosen for study with sunflower oil included to enhance the yield [24, 26]. A full factorial central composite design for five media components was generated using MINITAB 15 software and all the experiments were conducted in triplicate. A matrix of all experiments done in triplicate was framed with high and low coded values as +1 and −1, respectively. Six runs of central points were also included in the matrix. The factors and their high and low values are given in Table 1. The experimental design and the response variables are listed in Table 2. Two sets of each experiment were carried out.

The empirical form of the regression model developed is given by the following equation:

(1) Ŋ=A0+A1X1+A2X2+A3X3+A4X4+A5X12+A6X22+A7X32+A8X42+A9X1X2+A10X1X3+A11X1X4+A12X2X3+A13X2X4+A14X3X4

in which *A*_0_ represents the overall equation constant or the global mean and the subsequent regression coefficients for the other interactions are given by *A*_*i*_ (*i* = 1 to 14). The resultant *Ŋ* is the percentage emulsification index or the *E*_24_ index (*E*1) which is computed using the following formula:

(2) $$\begin{array}{c} \%E_{24}=\frac{\text{Height}\;\;\text{of}\;\;\text{emulsion}}{\text{Total}\;\;\text{height}\;\;\text{of}\;\;\text{the}\;\;\text{liquid}}\times 100. \end{array}$$

#### 2.3.4. Extraction and Purification of Bioemulsifier

500 mL of the optimised production media was sterilized and used for the production of bioemulsifier. To this, 0.25% sterile silicone oil was added as antifoaming agent. The production medium was inoculated with 2% seed culture and incubated on shaker incubator at 37°C and 90 rpm until the onset of deceleration phase (after 145 h). The cells were separated at 10,000 rpm for 10 minutes at 4°C, to obtain the spent broth.

The spent broth was subjected to a triple stage liquid-liquid extraction using equal volume of ice cold 2 : 1 chloroform : methanol solution and the organic phases were separated and pooled. The solvent was evaporated at room temperature (32°C ± 2°C) to concentrate the extract to 100-fold. This crude extract was further purified using reversed phase HPLC. Trifluoroacetic acid (TFA) (0.1%) in water and TFA (0.1%) in methanol were used as solutions A and B, respectively, and the components were eluted out at a flow rate of 0.5 cm^3 ^min^−1^ with solution B with a linear gradient from 30% to 100%. The elution pattern was monitored at 215 nm and the peaks were eluted out in fractions separately and each of them was tested for surface activity using oil spread assay and antifungal activity [27]. The oil spread assays were performed by comparing with SDS, Tween 80, and distilled water as controls. Lowry's total protein estimation method was used to evaluate the amount of bioemulsifier obtained and the result of media optimisation was evaluated by comparing the amount of bioemulsifier obtained from optimised media to that obtained using initial medium. Area under the peak method was used to evaluate and compare the yield of each peak, in both pre- and postoptimisation scenarios (M1 and optimised media fermentation).

## 3. Results and Discussions

### 3.1. Media Optimisation

#### 3.1.1. Preliminary Screening: One Factor a Time Method

**Figure 1:**
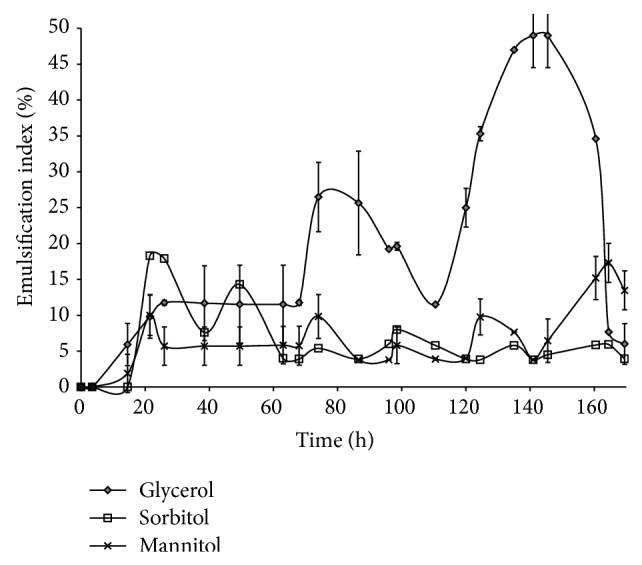
Selection of carbon source—one factor at a time.

**Figure 2:**
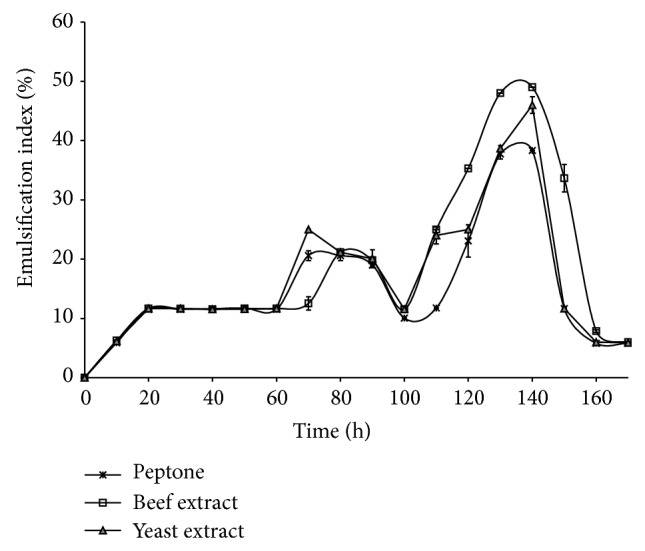
Selection of nitrogen source—one factor at a time.

Glycerol was found to be the best carbon source among all the three polyols that were taken for the study as it showed significant emulsification kinetics than the other counterparts (Figure 1). None of the inorganic nitrogen sources supported the growth of the organism, suggesting the fastidious nature of the organism. The effects of all the three organic nitrogen sources were comparable (Figure 2) but beef extract showed better results than the rest of the organic nitrogen sources. The production medium lacking microelements supported the growth of pak2310. But no significant emulsification was observed with the culture supernatant. Hence MS was found to be necessary for the bioemulsifier production.

It is clear from the results obtained that the inorganic salts in MS are not required to support the growth of the organism but play a very vital role in enabling the organism to produce the bioemulsifiers and hence should be present in the production media for bioemulsifiers. Certain trace elements like Mg, Ca, P, and Fe must have a role in the bioemulsifier production pathway.

Minimal salt containing glycerol and beef extract was then selected to be optimised for the bioemulsifier production. Previous studies indicate that addition of an insoluble and hydrophobic carbon source such as vegetable oil in an aqueous production media increases the production of bioemulsifiers [27]. Thus, the most common vegetable oil in the market, sunflower oil, was also taken as one of the factors for the optimisation of production media.

#### 3.1.2. Central Composite Regression Design: Statistical Approach

Experimental results, Table 2, were analyzed with MINITAB software and the regression analysis was performed at 95% confidence level. Student's *t*-test was performed to test the significance of the regression coefficients in the regression model (1). *T* value measures the size of the difference between the means. If the calculated *T* value is greater than the table *T* value the predictor is considered to be statistically significant. So, every time it is necessary to look into the *T* table to make interpretation of the results. On the other hand *P* value, which is calculated from *T* value, tells us the smallest *T* value leading to rejection of null hypothesis. Unlike *T* value, *P* value is easy to interpret. When *P* value is less than or equal to the confidence level, null hypothesis is rejected. For example, at 95% confidence level, if the *P* value is less than or equal to 0.05 the null hypothesis is rejected and we consider that the corresponding regression coefficient has significant effect on the response variable. On the other hand, if the *P* value is greater than 0.05 it is considered that the regression coefficient does not have statistically significant effect on the response variable.

From Table 3 it could be noted that the square interactions of BHB and BE and the paired interactions of BHB and BE; BHB and glycerol; BE and glycerol; BE and SF oil; and glycerol and SF oil (Table 3) have *P* value greater than 0.05. Thus, the corresponding regression coefficients are not statistically significant and can be removed from the model safely. Therefore, these terms were removed from the quadratic equation and the regression was repeated with the reduced model. Statistical parameters for the reduced model are given in Table 4.

From Table 4, it is evident that all factors included in the reduced model were statistically significant.

The reduced model that was developed after eliminating the insignificant interactions can be summarised as

(3) Ŋ=40−3.79X1−3.57X2−1.19X3−2.23X4−2.78X32+3.80X42+1.13X1X3−1.07X1X4.

The sunflower oil interaction (SF oil × SF oil) has a larger positive slope or coefficient value that indicates that the presence of SF oil in the media interacts favourably. Similarly the interaction of Bushnell Hass broth and glycerol also has a positive coefficient and hence their interactions are highly enriching for the production of bioemulsifier. These kinds of observations are not possible in conventional optimisation.

High *R* ^2^ (92.3%) value obtained suggested that the proposed model could explain the variation in the response variable associated with corresponding change in factor values.

In addition, in order to check the adequacy of the model, ANOVA was performed and the results are given in Table 5. The *P* values for main, interaction, and quadratic effects were all found to be less than 0.05. This observation further confirms statistical significance of all the regression coefficients included in the reduced model. *P* value for lack of fit at 95% confidence level was found to be 0.134 and also suggested that the reduced model was adequate to explain the variations in the response variable.

**Figure 3:**
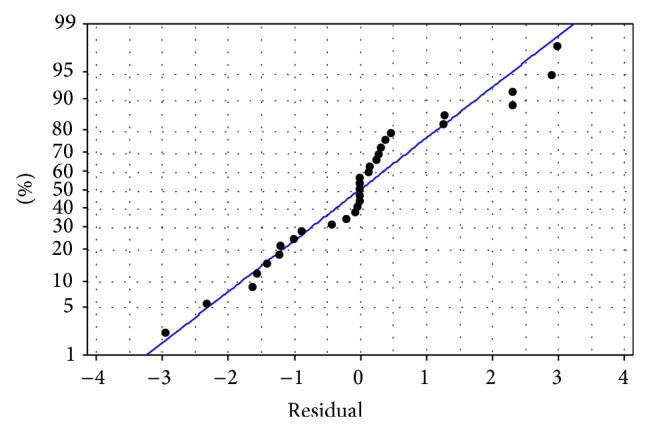
Normal probability plot for the experimental outcomes.

Adequacy of the model was further examined by residual analysis. The normal plot of residuals is shown in Figure 3. Almost all the values lie within the −2 to +2 range and spread evenly around the normal probability line. These observations confirm the adequacy of the model.

**Figure 4:**
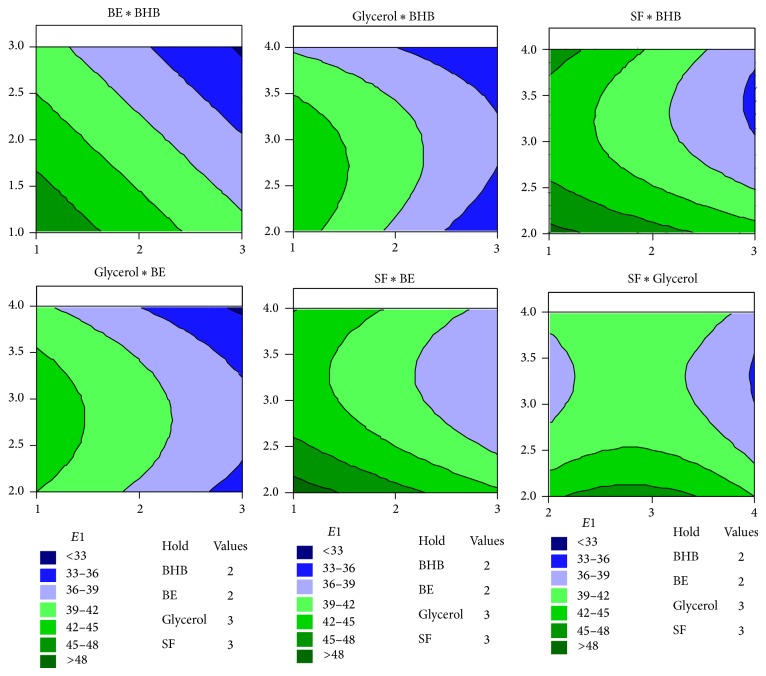
Contour plots for the experiments.

The contour plots (Figure 4) illustrate interaction between process variables graphically. Curved lines shown in the contour plots confirm that there is strong interaction between the variables. That is, the main effects of each factor included in the model depend on the levels of other factors. This observation was also consistent with the ANOVA results shown in Table 4. In conventional experimental designs, these kinds of observation are not possible. In addition, the contour plots help us to identify the optimum setting of variables to achieve maximum *E*1. In the contour plots shown in Figure 4, maximum value of *E*1 lies within the region shown in dark green color. Thus, maximum *E*1 could be obtained in regions where the values of BE, BHB, glycerol, and SF oil were 1, 1–1.5, 3, and 2–2.5, respectively.

Maximum *E*1 was obtained theoretically, by solving the quadratic equation (3) by inverse matrix method. Thus, a media with 1X concentration of BHB, 1% (w/v) BE, 2.8% (v/v) glycerol, and 2% (v/v) SF oil were deduced to be the optimum for the production of this bioemulsifier. The contour plots confirm the same graphically.

### 3.2. Extraction and Purification of Bioemulsifier

**Figure 5:**
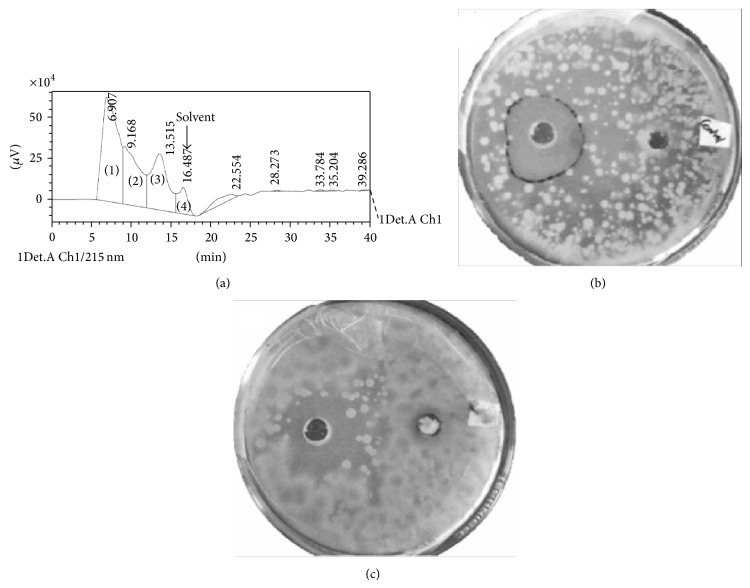
(a) HPLC analysis. Peak 1 was selected for analysis based on (b) antifungal activity of peak 1 at 3 days after incubation and (c) antifungal activity of peak 1 at 7 days after incubation.

**Figure 6:**
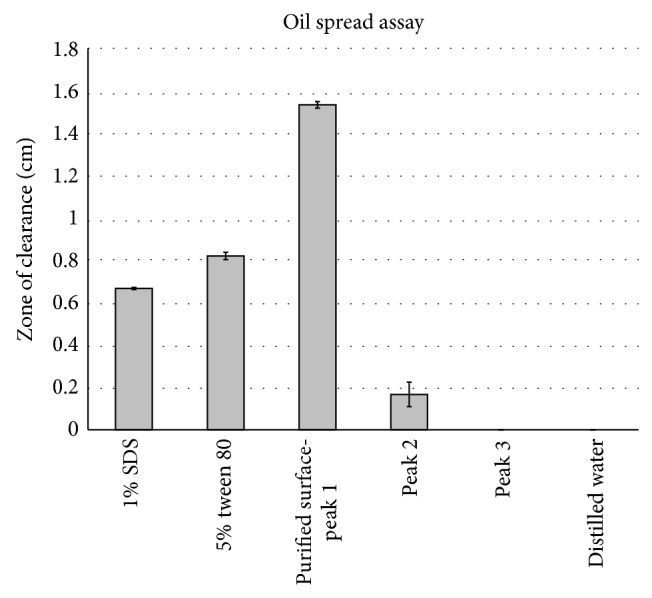
Oil spread assay for study of surface activity of different peaks along with standard surfactants and distilled water as control.

After 6 days of fermentation, the end of idiophase, as calculated from initial growth kinetics study for the standardised media, the exogenous bioemulsifier was extracted and purified. Similarly, a* Pseudomonas* strain has been reported to enter death phase by the end of the 7th day indicating that 6-day fermentation is not too long.* Bacillus *species are known to produce conjugated peptide surfactants like surfactins, iturins, fengycins, and bacillomycin. The lipid and water soluble, greasy, and pale yellow coloured bioemulsifier was obtained at the end of the triple stage liquid-liquid extraction and the fractions were pooled. The solvent was evaporated to a final crude extract volume of 10 mL from 500 mL at room temperature. The HPLC analysis yielded 3 peaks and the compound corresponding to the first peak eluted from 5 minutes to 9 minutes, constituting 42% of the total area under the chromatogram, was selected for further study as it had high antifungal property (Figure 5) and surface activity than the other two (Figure 6). The total protein estimation by Lowry on crude extract was evaluated to be 235.21 mg L^−1^ for crude extract before optimisation and 391 mg L^−1^ after optimisation. Using the area under the peak method, the bioemulsifier yield for optimised production media was 65.91% higher at 164.25 mg L^−1^ than that obtained with media of MS with 3% (v/v) glycerol and 1% (w/v) beef extract. In a similar experiment, Li et al. [28] have optimised media components to increase the yield of the pure biosurfactant by twofold.

## 4. Conclusion

Significant improvement in the yield of bioemulsifier was observed (~65%) under the new optimised conditions. The addition of a hydrophobic carbon source (sunflower oil) has indeed proven to be significant in yield improvement. Several other steps to improving the yield of the surfactants and also its recovery are in the laboratory pipeline and would form the basis of our future research.

## Figures and Tables

**Table 1:** High and low design of experiments—RSM media optimisation.

Factor	High	Low
MS	3X concentration	1X concentration
Beef extract	30 g#x2009;L^−1^	10 g#x2009;L^−1^
Glycerol	4% (v/v)	2% (v/v)
Sunflower oil	4% (v/v)	2% (v/v)

**Table 2:** Experimental design—RSM for media optimisation.

Run order	Point type	Block	BHB	BE	Glycerol	SF oil	*E*1 average
1	1	1	1	3	4	4	38
2	0	1	2	2	3	3	42.31
3	1	1	1	1	2	4	50
4	−1	1	2	2	2	3	37
5	1	1	1	1	4	4	46.15
6	1	1	3	1	2	4	34.61
7	1	1	3	1	4	4	40.44
8	1	1	3	3	4	2	35.71
9	1	1	1	3	2	4	41.4
10	0	1	2	2	3	3	40
11	−1	1	2	3	3	3	36
12	1	1	1	3	4	2	40
13	0	1	2	2	3	3	42.31
14	1	1	1	3	2	2	46
15	−1	1	2	2	4	3	36
16	−1	1	2	2	3	4	40
17	−1	1	3	2	3	3	36
18	−1	1	1	2	3	3	42.9
19	1	1	3	3	2	4	33.33
20	1	1	1	1	2	2	52
21	0	1	2	2	3	3	40
22	0	1	2	2	3	3	40
23	0	1	2	2	3	3	40
24	1	1	3	1	4	2	44.44
25	1	1	1	1	4	2	46
26	−1	1	2	2	3	2	46.15
27	1	1	3	3	4	4	28
28	1	1	3	3	2	2	37.33
29	0	1	2	2	3	3	40
30	−1	1	2	1	3	3	41.94
31	1	1	3	1	2	2	44.44

**Table 3:** Various factors and their interactions.

Term	Coefficients	SE coefficients	*T*	*P*
Constant	40.01	0.497	80.553	0.000
BHB	−3.79	0.395	−9.594	0.000
BE	−3.57	0.395	−9.045	0.000
Glycerol	−1.19	0.395	−3.009	0.008
SF	−2.23	0.395	−5.651	0.000
BHB ∗ BHB	0.21	1.039	0.197	0.846
BE ∗ BE	−0.28	1.039	−0.265	0.794
Glycerol ∗ glycerol	−2.75	1.039	−2.642	0.018
SF ∗ SF	3.83	1.039	3.685	0.002
BHB ∗ BE	−0.05	0.419	−0.121	0.905
BHB ∗ glycerol	1.13	0.419	2.707	0.016
BHB ∗ SF	−1.07	0.419	−2.552	0.021
BE ∗ glycerol	−0.77	0.419	−1.841	0.084
BE ∗ SF	−0.16	0.419	−0.393	0.700
Glycerol ∗ SF	0.43	0.419	1.026	0.320

**Table 4:** Significant factors and their interactions—refitted RSM.

Term	Coefficients	SE coefficients	*T*	*P*
Constant	40.00	0.470	85.122	0.000
BHB	−3.79	0.383	−9.892	0.000
BE	−3.57	0.383	−9.326	0.000
Glycerol	−1.19	0.383	−3.102	0.005
SF	−2.23	0.383	−5.826	0.000
Glycerol ∗ glycerol	−2.78	0.872	−3.184	0.004
SF ∗ SF	3.80	0.872	4.359	0.000
BHB ∗ glycerol	1.13	0.406	2.791	0.011
BHB ∗ SF	−1.07	0.406	−2.631	0.015

**Table 5:** ANOVA for significant interactions.

Source	DF	Seq. SS	Adj. SS	Adj. MS	*F*	*P*
Regression	8	691.14	691.14	86.39	32.76	0.000
Linear	4	602.24	602.24	150.56	57.10	0.000
Square	2	50.10	50.10	25.05	9.50	0.001
Interaction	2	38.80	38.80	19.40	7.36	0.004
Residual error	22	58.01	58.01	2.64		
Lack of fit	16	50.39	50.39	3.15	2.48	0.134
Pure error	6	7.62	7.62	1.27		

Total	30	749.15				

*S* = 1.624; *R* ^2^ = 92.3%; *R* ^2^ (adj.) = 89.4%; *F* = adj. MS factor/adj. MS error.
